# A Machine Learning Approach to Quantitative Analysis of Enamel Microstructure from Scanning Electron Microscopy Images

**DOI:** 10.1002/sstr.202400510

**Published:** 2024-12-25

**Authors:** Carli Marsico, Cameron Renteria, Jack R. Grimm, Juliana Fernandez-Arteaga, Donna Guillen, Dwayne Arola

**Affiliations:** Department of Materials Science and Engineering, University of Washington, Box 352120, Seattle 98195, WA, USA; Materials Science and Engineering Department, Idaho National Laboratory, Idaho Falls 98195, ID, USA; Department of Materials Science and Engineering, University of Washington, Box 352120, Seattle 98195, WA, USA; Department of Oral Health Sciences, School of Dentistry, University of Washington Seattle 98195, WA, USA; Department of Materials Science and Engineering, University of Washington, Box 352120, Seattle 98195, WA, USA; Science and Humanities Faculty, Institución Universitaria Digital de Antioquia, Medellín 050010, Colombia; Materials Science and Engineering Department, Idaho National Laboratory, Idaho Falls 98195, ID, USA; Department of Materials Science and Engineering, University of Washington, Box 352120, Seattle 98195, WA, USA; Department of Oral Health Sciences, School of Dentistry, University of Washington Seattle 98195, WA, USA; Department of Mechanical Engineering, University of Washington Seattle 98195, WA, USA; Department of Restorative Dentistry, School of Dentistry, University of Washington, Seattle 98195, WA, USA

**Keywords:** bioinspirations, enamel, image analyses, machine learning, machine visions, scanning electron microscopies

## Abstract

Dental enamel, the outermost tissue of mammalian teeth, must withstand a lifetime of wear and cyclic contact. To meet this demand, enamel possesses a combination of high hardness and resistance to fracture, properties that are typically mutually exclusive. The impressive damage tolerance has been attributed largely to decussation of the enamel rods, the principal unit of its microstructure. As such, enamel is inspiring the design of next-generation structural materials. However, quantitative descriptions of the decussated enamel rod microstructure remain limited due to challenges encountered in applying computed tomography and in acquiring quality images appropriate for traditional digital processing methods. Here, a machine learning segmentation method is applied to images of the enamel obtained using scanning electron microscopy to support quantitative analysis of the microstructure. A pretrained convolutional neural network is used to expand the input training image dataset to allow the training of a random forest classifier, which ultimately segments the image with a very small training set (*n* = 3 images). A validation of this segmentation method is presented, in addition to its application to calculate relevant microstructural parameters for images of tooth enamel from selected mammalian species. The methodology applied here is equally applicable to other hard tissues.

## 1. Introduction

Mineralized biological tissues possess complex microstructures that are recognized for their ability to bestow combinations of mechanical properties that are often mutually exclusive, including hardness and resistance to fracture.^[1–3]^ The microstructures of these materials are key to achieving the required durability over a wide variety of functions. As such, highly mineralized tissues have become potent sources of design inspiration for next-generation biomaterials and bioinspired structural materials. The enamel of mammalian teeth is one tissue in this category.

Tooth enamel exhibits a complex hierarchical microstructure that has received substantial research attention.^[4–7]^ The foundational unit of enamel is the hydroxyapatite crystallite. These crystallites are aggregated into an assembly to form rods, which are ≈5 μm in diameter. Between each rod, the interprismatic enamel consists of a thin layer (about 0.5 μm thick) with higher organic content and lower relative packing density. The rods are further organized into two enamel types, largely based on their orientation. The radial enamel, generally located toward the outer surface of the tooth, consists of rods that are oriented parallel to one another and uniformly toward the outer enamel surface. Located closer to the dentin enamel junction (DEJ), decussated enamel consists of rods that are organized in woven bands. The final level of hierarchy (the “Schmelzmuster”) refers to the distribution of the radial and the decussated enamel through the thickness of the enamel crown. Detailed descriptions of these levels of hierarchy are outlined elsewhere in the literature.^[6–8]^

The fundamental unit of the enamel microstructure that is most evident in microscopic evaluations is the enamel rod. These are cylindrical formations (often regarded as prisms) that extend from the DEJ to the tooth’s outer surface.^[9]^ One of the most distinctive features of the enamel microstructure is decussation, where enamel rods are distributed in a woven assembly that traverses through the tissue.^[10,11]^ The complicated arrays of woven rods in the decussated enamel serve to deflect advancing cracks, thereby promoting what is regarded as “rising R-curve” behavior, reflecting the increase in crack growth resistance with an increase in crack length.^[12–17]^ Indeed, this decussated arrangement of rods enhances the tooth’s overall resistance to fracture and plays a key role in its ability to withstand the forces encountered during chewing.^[18]^

While the decussation patterns in human enamel have received considerable attention (e.g.,^[5,19–21]^), the decussation pattern of rods in other mammals has not. Despite the implications to evolution, the present understanding of decussation, especially concerning potential correlations with bite forces and feeding behaviors, remains sparse. In fact, due to difficulties associated with imaging enamel and the degree of microstructural complexity present, there is very limited quantitative information available on the decussated enamel microstructure overall. Previous research within our group has identified differences in the mechanical behavior of enamel across species with variations in diet, body mass, and bite force.^[22]^ These differences in mechanical properties could be a result of structural adaptations affecting the underlying microstructure, which warrants further investigation.

In the past decade, there has been substantial progress in characterizing the microstructure of mineralized tissues by scanning electron microscopy (SEM) and focused ion beam milling. Reviewed by House et al.^[23]^ this approach provides the capability for evaluating the complex 3D structure of tissues, including tooth dentin and enamel, and contributing new understanding. Rod topography, often exposed by cross sectioning, is readily observed in individual SEM images. Through serial sectioning and imaging, SEM data can be nested layer-wise to yield a tomographic reconstruction, but the volume analyzed is limited to tens or 100s of cubic micrometers. There have also been substantial contributions to understanding the 3D structure of enamel at the nanoscale using atom probe tomography^[24]^ and polarization-dependent imaging contrast mapping.^[5]^ However, there is a significant lack of complementary characterization at the next level of structural hierarchy, specifically at the level of rods and their pathways. If the purpose of quantitatively describing the microstructure of enamel is for mimetic endeavors and the development of structures capable of achieving new levels of damage tolerance, then a quantitative description of the decussated microstructure is key. Recent work has been performed using synchrotron phase contrast imaging of mammalian enamel and complimentary characterization to elucidate the rod distributions and decussation patterns.^[25,26]^ While that approach is robust, it requires access to an advanced light source, which ultimately limits the number of samples that can be analyzed.

In order to successfully mimic the structure–property relationships observed in enamel, it is imperative to develop high-throughput and robust methods for quantitative analysis. While it is well known that enamel rods guide crack growth in enamel, minimal progress has been made in developing methods to quantitatively assess the rod orientation, which is considered a key contributor to crack growth toughening.^[15,27]^ While recent studies have found differences in the mechanical properties across selected mammalian species, the quantitative understanding relating enamel microstructure, function, and evolutionary adaptations is absent.^[22,27–29]^ To develop viable digital models of enamel that can support the manufacture of damage tolerant engineering materials, the microstructure of enamel microstructure must be understood quantitatively. To this end, the objective of this investigation was to apply a machine learning segmentation method (i.e., machine vision) to SEM images of enamel to explore the opportunity for performing quantitative analyses of the decussated microstructure. Specifically, this investigation focuses on the enamel rod pitch angles, which are key to crack growth toughening and have been most elusive in conventional imaging.

## 2. Background

The first step to any automated quantitative analysis of microstructure through image analysis is segmentation, that is, the process by which an image is processed (e.g., binarized) so that the features of interest can be separated from the background and analyzed.^[30,31]^ Segmentation is traditionally performed using a series of filters, thresholding, and morphological operations.^[30]^ Once the important features are identified, more sophisticated algorithms, such as watershed, can be used to separate overlapping objects so that quantitative parameters associated with geometry such as diameter, solidity, and sphericity can be calculated for each individual object.^[30]^ However, these analyses require that a good initial segmentation of the image is achieved first, which in turn relies on an image with a good signal-to-noise ratio and clear boundaries between the objects of interest and the background.^[30]^ Modern software interfaces allow for comparatively quick and effective segmentation of most engineering materials,^[30,31]^ but this is not the case for enamel.

Unlike engineering materials where most objects of interest are relatively sparsely embedded within a matrix material or delineated by clear boundaries with significant contrast, enamel possesses neither of those characteristics.^[31]^ Imaging of enamel is generally complicated by poor contrast, an issue attributed to its density and uniformity in composition. Enamel is ≈96% calcium phosphate-based mineral with the balance consisting of water and organic matter,^[32]^ which limits the viability of chemical contrast between rods. Topographical contrast can be introduced on a polished cross section by application of an etchant, which provides contrast between rods but also nanocrystals. However, the fine structure of the nanocrystals within each rod hinders the success of traditional processing techniques to identify individual rods,^[31]^ and so an alternative method for enamel segmentation is required.

The recent application of machine learning to image analysis and segmentation has resulted in approaches that are dependent solely on the ability to generate training data rather than the specific content of the image.^[33]^ One of these methods utilizes a pretrained convolutional neural network (CNN) to provide additional quantitative input about each pixel present in the image of interest.^[33–35]^ CNNs are trained on large sets of general images with a variety of subjects (cats, dogs, vehicles, etc.).^[33]^ The result is a network that is excellent at identifying patterns and textures within an image regardless of the subject.^[34]^ When an image is processed through the pretrained network, it produces a large set of additional parameters about the image.^[33,34]^ These additional parameters are then used as input for a random forest (RF) classifier (or other classifier) to categorize each pixel as either background or feature.^[33,34]^ The benefit of this approach is that a model can be trained to segment any kind of image, provided that an adequate training set is available.^[33]^ Once the model is trained, it can be used to automatically segment many images quickly without the need for expensive computing resources.

Although the subject matter in images of enamel microstructure is not as simple, it was hypothesized that such an approach might be useful for segmenting enamel rods, enabling the rapid analysis of enamel microstructures from a statistically relevant dataset. Once images are segmented, calculating elementary quantitative parameters for each rod such as diameter, position, incident angle with the imaging plane, and total area fraction of rods becomes trivial, enabling a more comprehensive description of enamel. Here, the application, optimization, and validation of this methodology for use with enamel SEM images is described.

## 3. Experimental Section

### 3.1. Preparation of the Training and Validation Datasets

**Figure 1.**
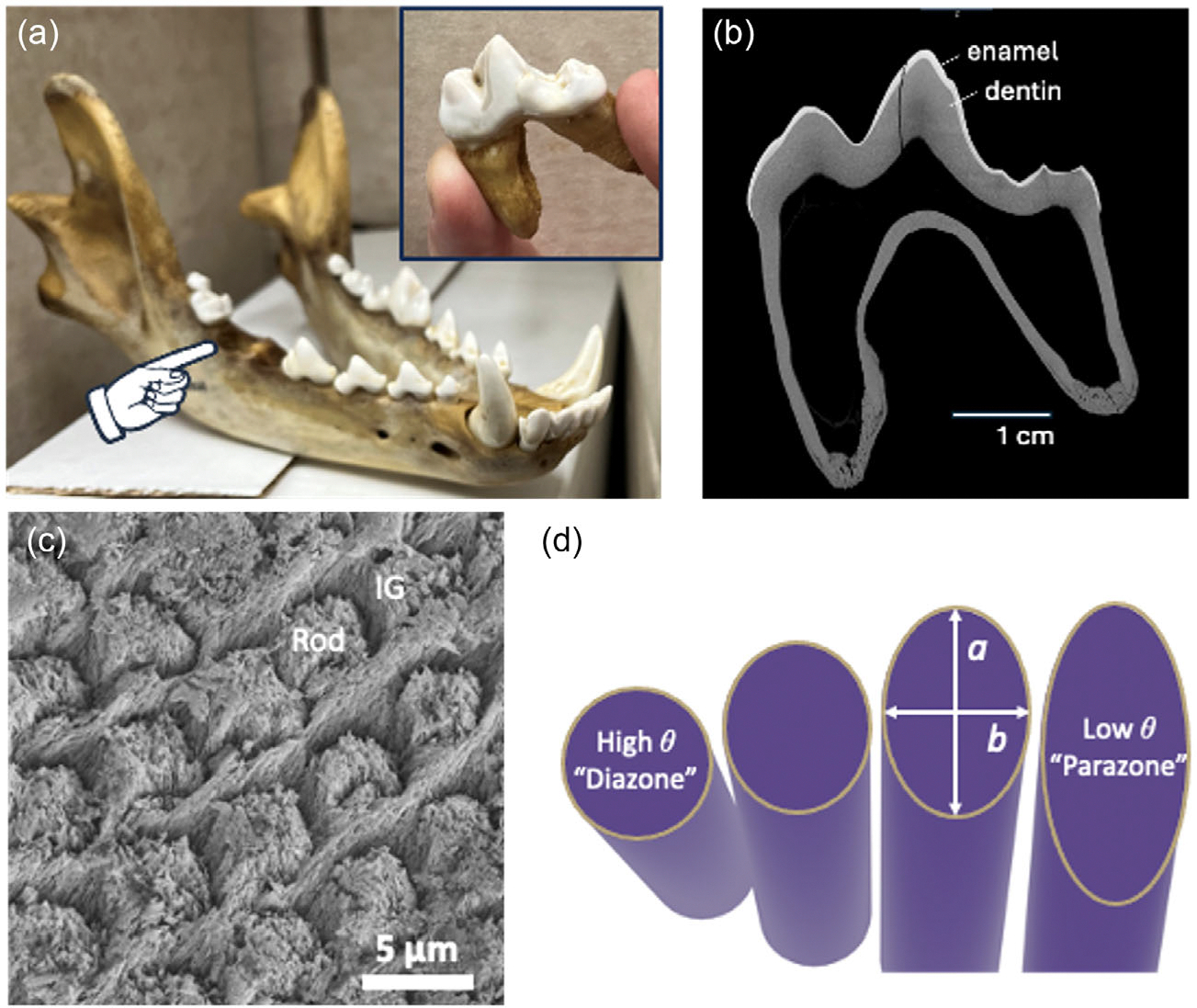
Details of the experimental evaluation involving the evaluation of enamel. a) Acquiring a wolf tooth, b) locations of dentin and enamel, c) SEM image of diazone rods in a polished and etched buccal-lingual cross section. The intergranular (IG) phase complicates traditional segmentation of the rods. d) Pitch *θ* is measured from the ratio of *a* & *b* of a fit ellipse drawn on the perimeter of the rod when sectioned by the imaging plane.

Various SEM images of enamel were used in this investigation. The images were acquired by our group over several years of research focused on comparing the mechanical properties of tooth enamel across different carnivorous and omnivorous species.^[22,25]^ In general, the images were taken from buccal-lingual sections of molar teeth, as evident in Figure 1a,b. The preparations involved sectioning the teeth axially, mounting in epoxy, polishing to a mirror finish, etching the polished surface with phosphoric acid, and then coating with gold-palladium to allow for good imaging of the microstructure. Details of these procedures have been reported elsewhere.^[22]^

In most cases, the SEM imaging was performed on a XL30 system (FEI) with an accelerating voltage of 10 kV and a spot size of 1. Micrographs were taken primarily at 500x magnification and resolution of 640 × 520 pixels, resulting in a field of view of ≈244 × 144 μm.

A total of *n* = 3 images were manually segmented to use as training images and a fourth image (*n* = 1) was segmented to use as a validation image to assess the success of the model. The training images consisted of two SEM images of enamel from a lion molar (*Panthera leo*, African lion) and one SEM image of enamel from a black bear molar (*Ursus americanus*, American black bear). Both teeth were obtained in the dehydrated condition from the Burke Museum of Natural History and Culture (Seattle, WA). The validation image consisted of one image taken from a lion tooth. The lion and black bear images were chosen for the training datasets because of the high quantity and quality of the SEM images that were obtained for these two species. The decussation patterns and rods exhibited by these two species were representative of the range in those features that our team visualized considering all animals. As such, no bias was expected.

Manual segmentation was performed in Apeer (Carl Zeiss Microscopy GmbH, Jena, Germany) by painting individual pixels by hand to label the visible enamel rods. This process resulted in four raw images with four associated binary masks each with ≈300–600 rods labeled.

In the process of optimizing the segmentation model, it was found that the accuracy could be increased by applying some augmentation steps to the training images. Augmentations are simple modifications applied to both the input images in the training dataset and their corresponding maps to help ensure that the model is generalized. In semantic segmentation, augmentation must be applied conservatively since gross distortions to the training images can result in a model that is far less accurate than a model trained without augmentation. Here, only left-right and top-bottom flip augmentations were applied. This expanded the training dataset to a total of nine images: 1) the original image, 2) the original image reflected left to right, and 3) the original image reflected top to bottom for each of the three training images.

### 3.2. Model Architecture

The segmentation model consisted of two portions, including 1) the pretrained CNN model whose purpose was exclusively to perform feature expansion and 2) the RF classifier, which sorted pixels into either rods or background based on the input parameters. Only the RF model was trained; no CNN model training was performed. VGG16 was used as the pretrained CNN.^[36]^ VGG16 consisted of 16 sequential convolutional layers in five blocks each with varying numbers of filters within them.^[36]^ Each block was separated by a max pooling layer; however, these were omitted from the segmentation model as were the activation layers since VGG16 was exclusively used to perform parameter expansion. The precise layers and the number of layers used were optimized as described in Section 3.3.

Raw training images were passed through VGG16 to expand the input features from one parameter per pixel to between 64 and 4224 parameters per pixel, depending on the number of VGG16 layers used. The expanded parameters from all three of the training images were combined and used as the input training set for the RF model. Likewise, the binary maps for all three training images were combined to generate the key for the training set upon which to tune the classifier model. Standard RF classifier parameters were used,^[37]^ and the number of trees varied from 50 to 150. Optimization was also performed on the RF classifier as described in Section 3.3. All training was conducted on the Sawtooth high-performance computing enclave at Idaho National Laboratory (Idaho Falls, ID) using one dedicated graphical processing unit. Training for all RF classifiers was performed until convergence was achieved.

### 3.3. Model Optimization

Optimization was performed on both parts of the segmentation model individually. The best specific VGG16 layers to use and the number of layers that yielded the best segmentation were identified by evaluating test models on all possible combinations of layers and the number of layers within the VGG16 architecture. The same RF classifier with 50 trees was used for each layer combination and the accuracy of each model was calculated on the validation image. Since there was a relatively low and finite combination of layers, and an RF classifier with only 50 trees and three training images was used, training each test model required only 10–30 min. This ensured that a brute force approach was not overly resource intensive to preclude its use. The layer combination that produced the highest accuracy was used in all the following analyses performed.

Optimization of the RF classifier was performed using a Bayesian approach to tune the RF hyperparameters.^[38]^ Model accuracy was calculated on the validation image with the VGG16 layer optimization. The best set of hyperparameters identified only produced marginally improved accuracy, so ultimately the RF classifier was simplified to a model trained with the standard parameters composed of 150 trees. A fivefold cross validation was also used to evaluate the model’s ability to perform when given new data.

### 3.4. Quantitative Analysis

Following segmentation, several microstructural parameters of interest were selected to be calculated from the data. The primary parameter of importance to bioinspired efforts is the pitch angle of the enamel rods (Figure 1c,d), which represents the out-of-plane tilt.^[25]^ Since cracks extended along the rod boundaries, their pitch orientation represented a crack deflection angle, with higher values resulting in more tortuous paths. Pitch can be calculated from elliptical cross sections of rods (Figure 1d) according to^[39]^

(1) $$\theta =\sin^{-1}\left(\frac{b}{a}\right)$$

where *θ* denotes pitch in degrees, *b* denotes the minor axis of the ellipse fit to the rod object, and *a* denotes the major axis of the ellipse fit to the rod object.

A mild despeckle operation was performed on the segmented image output by the segmentation algorithm to remove any objects smaller than 20 pixels in total area.^[30]^ Then, ellipse parameters of fit (i.e., *a*, *b*, and the orientation of the major axis of the ellipse), object diameter, area, solidity, and position were all calculated using conventional image analysis techniques included in the Scikit Image package for python. This processing produced a set of parameters for each object within the segmented image, which were then used to calculate the pitch for each object according to Equation 1. The terms “parazone” and “diazone” have often been used in the literature to refer to bands in the decussated enamel with prevailing rod trajectories in plane or out of plane, respectively. Diazone rods were designated as any rod with a pitch value greater than 25°. Additionally, only rods with a fit ellipse minor axis length (b) less than 5.5 μm were considered, since the minor axis length was indicative of the rod diameter, which was around 5 μm in the tooth enamel of most species. The assessment of rod objects according to the diameter helped to identify rods that merged with adjacent rods resulting in an unrealistic segmentation. Finally, a maximum area of 70 μm^2^ was stipulated to filter objects that included multiple merged parazone rods. The in-plane angle, or yaw, was also assessed for each rod object as the angle between the major axis of the fit ellipse and the vertical axis of the image.^[39]^ These estimates were obtained using conventional image analysis techniques.^[30]^ To assess the variation in pitch as bands transition from one type to another, an analysis direction across bands was defined as being either parallel to the DEJ (when visible in the micrograph) or perpendicular to the average yaw of parazone rods.

### 3.5. Validation and Statistical Analysis

The quality of various segmentation models was calculated from the validation image. The accuracy was calculated as the percentage of correctly classified pixels (feature or background) divided by the total number of pixels in the image. This metric was analogous to the intersection over union (IoU) metric more commonly used to quantify image classification accuracy.^[40]^

The accuracy of the pitch calculations resulting from the overall segmentation was assessed by comparing the overall pitch maps calculated from the validation image and the segmented image as well as the specific pitch values calculated from a small subset of specific rods, which could be identified in both the validation and segmented images. A registration process was conducted, which began by identifying rod objects within the algorithm-segmented and ground truth image that shared centroid coordinates (±5 pixels). Then the pitch value from the object in the ground truth was plotted versus the pitch value in the object from the model output. This plot should produce a straight line for a perfectly accurate segmentation. Through the coefficient of determination (*R*^2^), a quantitative assessment of the segmentation quality could be calculated using the numpy package in python. An *R*^2^ greater than 0.90 was considered as significantly similar. Finally, the segmentation model was tested on additional 500x images of tooth enamel obtained from mammal species not represented in the training dataset, as well as images at different magnifications obtained from a variety of species.

## 4. Results and Discussion

### 4.1. Model Optimization

**Figure 2.**
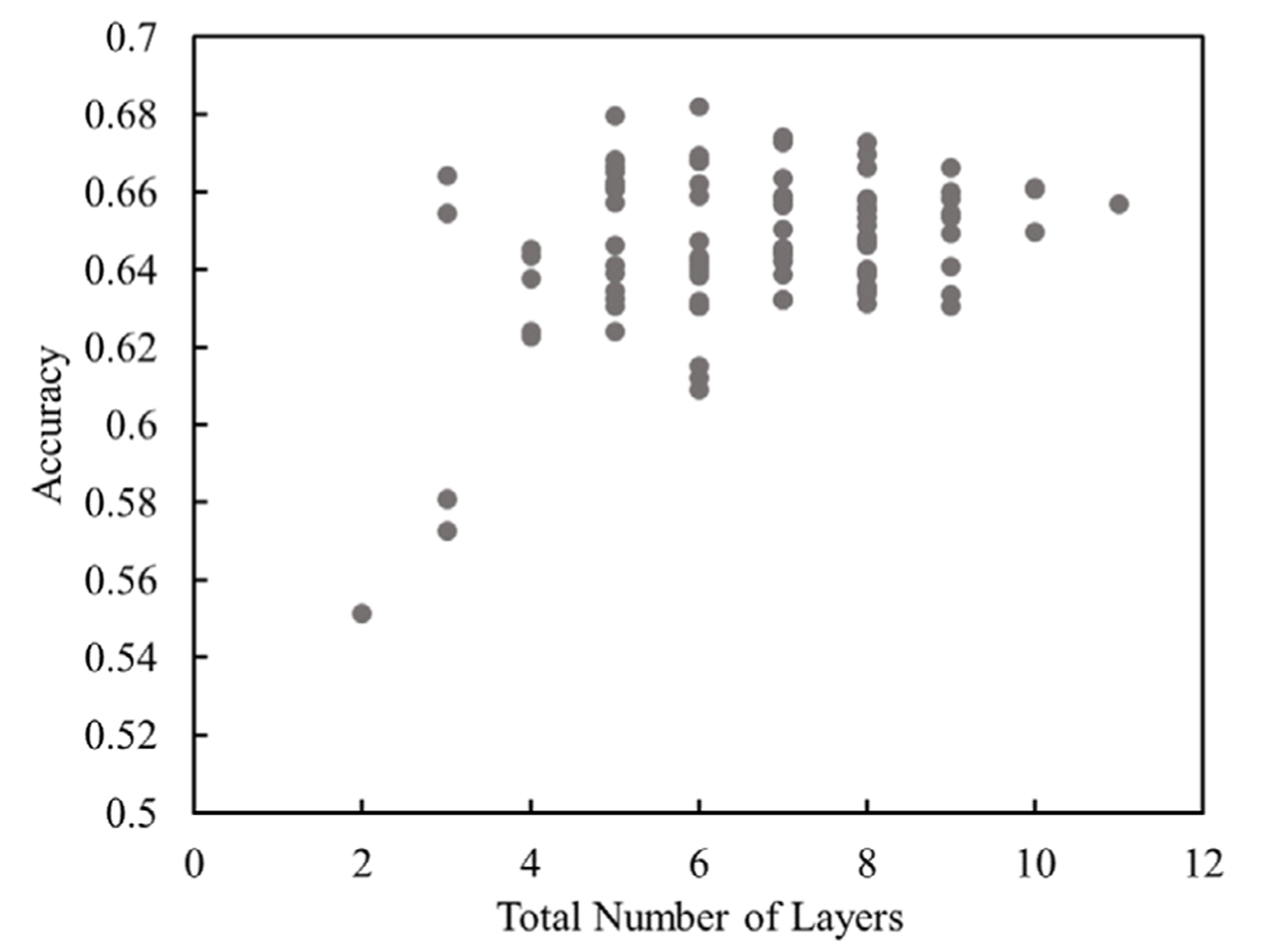
Plot of model accuracy as a function of the number of input layers used from VGG16. The accuracy is based on the use of *n* = 3 training images and a RF model of 50 trees.

Figure 2 shows the accuracy of 100 random combinations of VGG16 layers versus the number of layers used. As evident from the response, accuracy increased with the number of layers up to a point, beyond which additional layers resulted in a decrease in accuracy as expected. Too many parameters can result in the fitting of noise that does not translate to greater accuracy on the validation dataset, which is not included during training. Conversely, too few parameters make it difficult to identify the patterns of interest. This transition occurred between five and seven VGG16 layers.

The highest model accuracy achieved overall, that is, for any test segmentation model (based on *n* = 3 training images and an RF model of 50 trees), was 68.9%. The accuracy with these specific layers (additional details provided in the Supporting Information) increased to 71.6% when the number of trees used in the training model was increased to 150. Further improvements in accuracy were not achieved with further increases in the number of trees, which is typical for RF classifiers since too many trees result in an extremely large number of training parameters and long training times for which an optimum is not easily identified.^[41]^

Bayesian optimization of the RF classifier hyperparameters resulted in a 1.5% increase in model accuracy over the standard test model. But this increase was not constant with variations in VGG16 layer combinations. Furthermore, simply increasing the number of trees in the RF classifier resulted in better accuracy than the hyperparameters identified from the Bayesian hyperparameter tuning. This indicates that there was too much noise in the system for the Bayesian optimization algorithm to perform accurately. It is possible that the optimization entered into a local extreme and was consequently not able to identify a better set of hyperparameters than was achieved with standard hyperparameters and 150 trees. Nevertheless, it was decided to continue with optimization using exclusively the “optimum” VGG16 layer combination and standard RF classifier hyperparameters and 150 trees.

**Figure 3.**
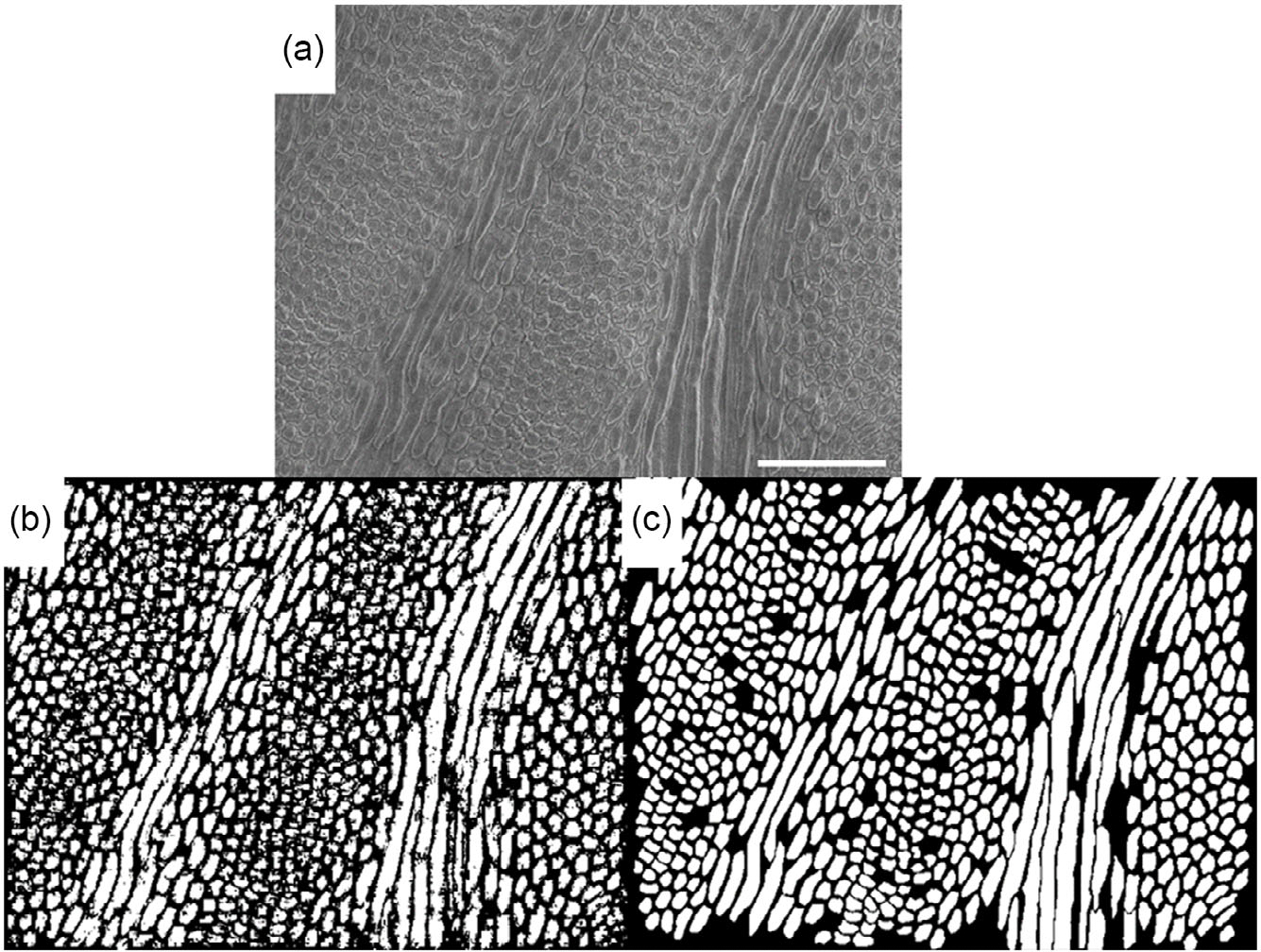
Comparison of a) the raw SEM image of decussated enamel, with b) the algorithm segmented validation image, and c) the ground truth manually segmented validation image. The scale bar in (a) =50 μm and is the same scale used in (b) and (c). The comparison involves a sample size of (*n* = 1), which does not support probability value estimation or statistical test.

Next, image augmentation was applied to expand the dataset further and ensure that the segmentation algorithm remained generalized to images of the enamel microstructure regardless of the orientation in which they were captured.^[42]^ Applying the left-right and top-bottom image transposes and retraining the previously optimized model on the expanded training set increased the accuracy by 7% (78.6% total accuracy). Implementing fivefold crossvalidation on the same model architecture and training set resulted in a slight decrease in accuracy (77.7%) but a much faster training time (31 min vs. 166 min with no crossvalidation). However, since the training time was still comparatively short compared to many other machine learning approaches, this reduction in training time wasn’t considered an advantage at the cost of a slight decrease in accuracy. In the final optimized segmentation mode, no crossvalidation was included. Figure 3 shows a comparison of the most accurately segmented validation image (Figure 3b) and the ground truth manually segmented map (Figure 3c). While the output of the segmentation model is noisy, it clearly identifies rod features in both the parazone and diazone bands.

While various optimization protocols were applied to improve the segmentation model as much as possible, there are many additional optimization techniques available.^[43]^ More constrained tuning of the RF hyperparameters using the optimal VGG16 layers identified earlier may lead to a set of parameters that provide an improvement over the standard set of RF hyperparameters with 150 trees.^[38]^ The pretrained CNN used to expand the data set prior to training the RF classifier can also be changed. There are many pretrained CNNs available, that is, VGG19, Xception, Resnet50, etc. Each of these has slightly different architectures and layer weights, which make them slightly better at detecting certain types of images.^[33,34,36,44]^ However, it is difficult to identify the CNN that will perform best on a given image set without performing additional testing in a model. While possible, it is unlikely that using a different pretrained CNN would garner a substantial increase (>5%) in segmentation accuracy since most recent pretrained models perform similarly according to comparisons in the literature.^[44]^ Since satisfactory results were achieved with the currently optimized model, no further optimization was pursued with the enamel images. Further optimization is not likely to provide substantial increases in accuracy.

### 4.2. Quantitative Validation

**Figure 4.**
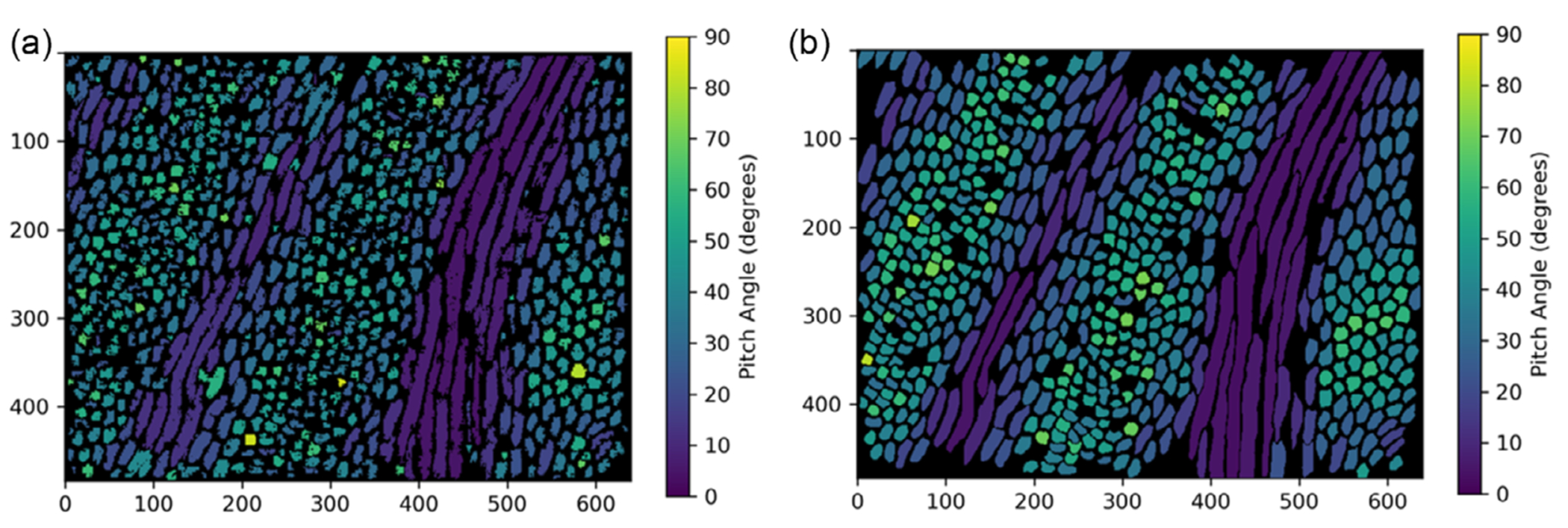
Comparison of the pitch values calculated for each rod object through color maps for the image. a) The algorithm segmented validation image, and b) the ground truth manually segmented validation image. The X and Y axes show the pixel coordinates, and the color bar indicates the pitch value. These images consist of *n* = 512 rods. Comparisons are shown in Table 1 and Figure 5.

Figure 4 compares the pitch maps generated from the algorithm segmented validation image with the ground truth validation map. The magnitude and distribution of pitch angles in this figure clearly show the distinct diazone and parazone bands.

Except for a few rods that merged with adjacent rods, the magnitude and distribution of pitch angles roughly match that of the ground truth validation map. Pitch angles gradually transition from low-angle values in the parazone bands to higher angles in the diazone bands with an overall average of ≈40°. This matches the general trend in pitch values that have been observed previously.^[25]^ Table 1 lists the pitch values for a small subset of rods that could be clearly identified in both the algorithm segmented and ground truth maps. (Figure S1, Supporting Information, shows the location of these rods within the validation image.)

**Figure 5.**
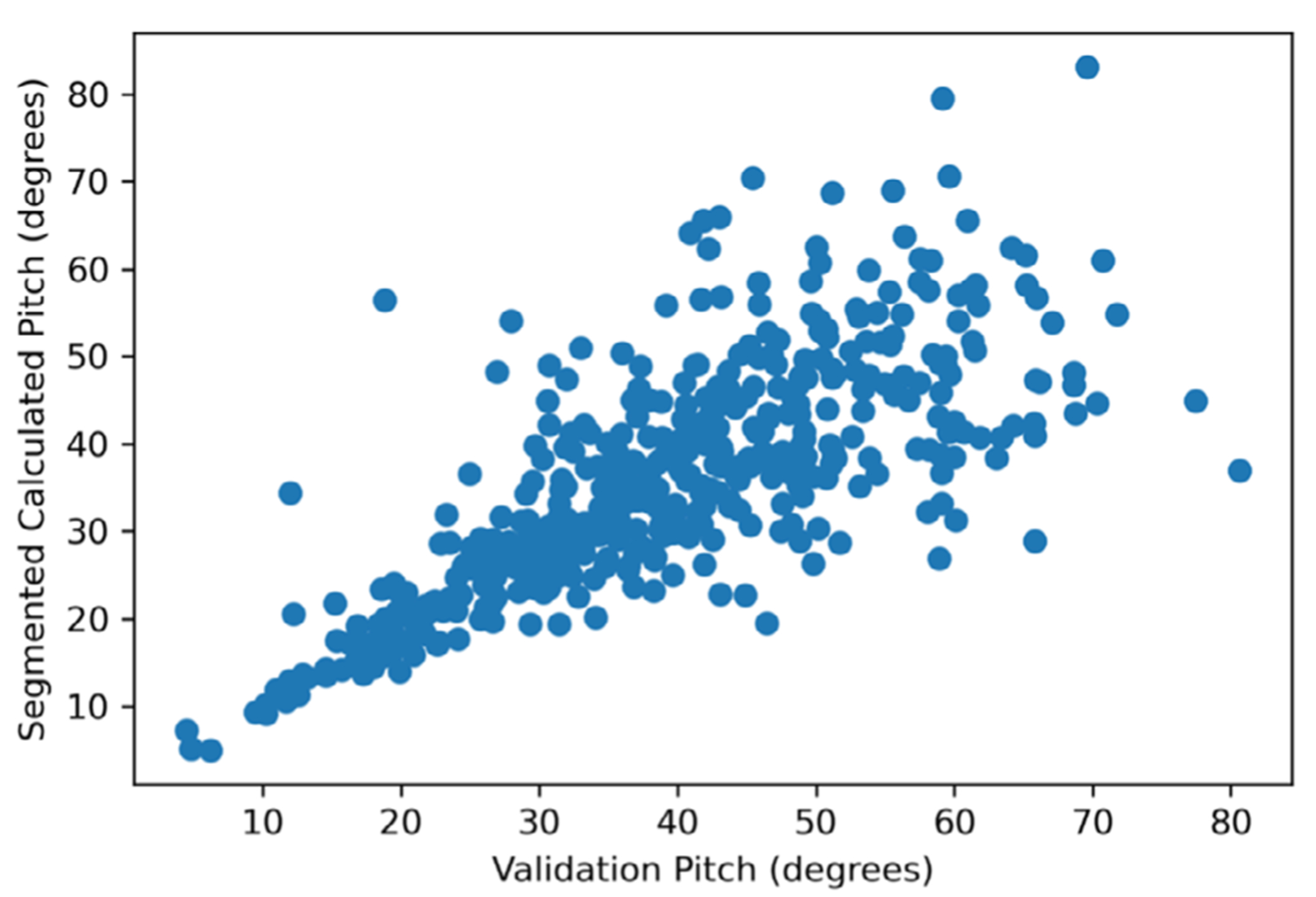
Predicted pitch value plotted as a function of the ground truth pitch *n* = 461 successfully registered rods out of a total in the image space of 512. The plot roughly follows the *x* = *y* line as expected; however, there is more spread in the data at larger pitch. A registration correlation of the *R*^2^= 0.572 was achieved, compared to a perfect registration of *R*^2^= 1.0.

Figure 5 shows the pitch values of the registered rods from the ground truth image versus the values from the algorithm segmented image. In total, 461 rods out of the 512 rods present in the ground truth map were successfully registered (≈90%). Admittedly, the *R*^2^ = 0.572 is much lower than preferred. Generally, a favorable *R*^2^ value is greater than 0.9.^[45]^ However, given that there is likely some error in the registration process and in the manually segmented map, which was subjective, a lower *R*^2^ value than desired was not unsurprising. The segmentation algorithm appears to struggle most at identifying rods with larger pitch values correctly. For those rods, very small changes in the major and minor fit ellipse axes can have a comparatively large effect on the calculated pitch value.

**Figure 6.**
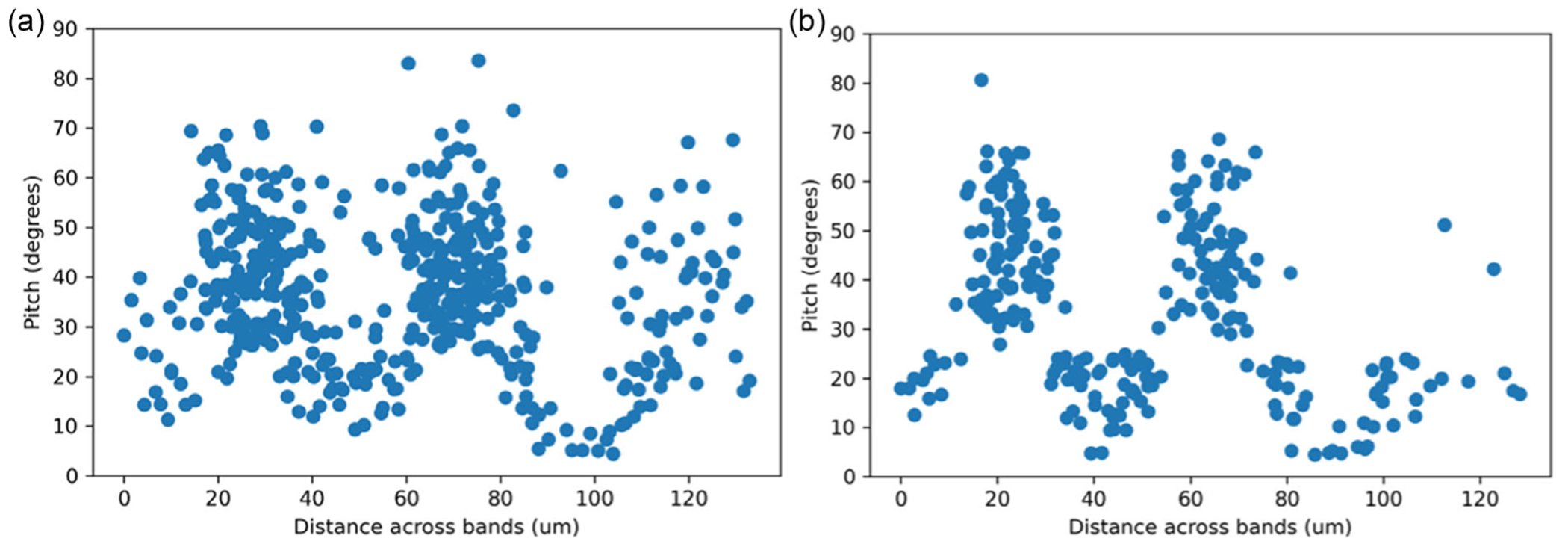
Comparison of the pitch values as a function of distance across the bands for a) the algorithm segmented validation image and b) the ground truth manually segmented validation image. These figures consist of *n* = 512 data points from the rods presented in Figure 4. This is a visual comparison that does not support probability value estimation or statistical test.

Finally, the variation in pitch across a selected range of bands was compared. Figure 6 shows the crossband pitch variation for both the algorithm segmented and manually segmented images. As evident from this image, the pitch variations across the band are similar for both images. While the band periodicity and pitch magnitude are similar, there is more spread in the algorithm segmented data, as expected. The caveat with 2D image analysis is that the alternating positive and negative polarity of the diazone bands described in another study^[25]^ is obscured. The graphs in Figure 6 represent essentially the absolute value of the diazone (D) pitch angle, that is, the range in pitch angles is from 0 to +90° (representing +D and |–D|, rather than +D and –D). With that recognized, quantitative analysis can be conducted appropriately.

It is important to highlight that the magnitude of the variation in pitch observed from the SEM images in Figure 6 is greater than that recently observed for the enamel of the lion scans obtained using synchrotron phase contrast micro computed tomography.^[25]^ However, recognizing that the angles observed from the manual and algorithm segmentation were in agreement, it was concluded that this was not a result of inaccuracy in the segmentation method. Rather, the differences are more likely attributed to inconsistencies in the exact location within the tooth from which the images were extracted or the inability to confirm if the buccolingual section captured in the SEM images was perfectly perpendicular to the band axis. Both differences could lead to changes in the estimated pitch magnitude. Further work is warranted to address this concern.

Based on the results from these validation exercises, it was concluded that the automatic segmentation and pitch calculation algorithm for enamel successfully identified rod features and determined the associated pitch values with acceptable accuracy. Although the segmentation introduces some additional noise into the data, this is expected. The effort consists of automating a visually driven segmentation task that is difficult to achieve even manually for a highly skilled operator. There is also noise present from the manually segmented training maps where errors in the initial rod segmentation were present, which may be confounding the RF classifier during training. Regardless, the substantial increase in quantitative data acquired using this method over traditional/manual methods far outweighs the drawback of introducing a small degree of additional noise to the data.

### 4.3. Application to other Images and Species

The training dataset only included images of tooth enamel from the lion and black bear. Consequently, there was some concern that the segmentation model was trained such that it overfit to the specific patterns observed in enamel from those two species. To determine whether overfitting had occurred, additional images from the tooth enamel of alternative species were processed via the segmentation algorithm, including enamel from snow leopard (*Panthera uncia*), wild African dog (*Lycaon pictus*), and raccoon (*Procyon lotor*).^[46]^ Some of these images were obtained at different magnifications since 500x magnification images were not available for the enamel microstructure of all species.

**Figure 7.**
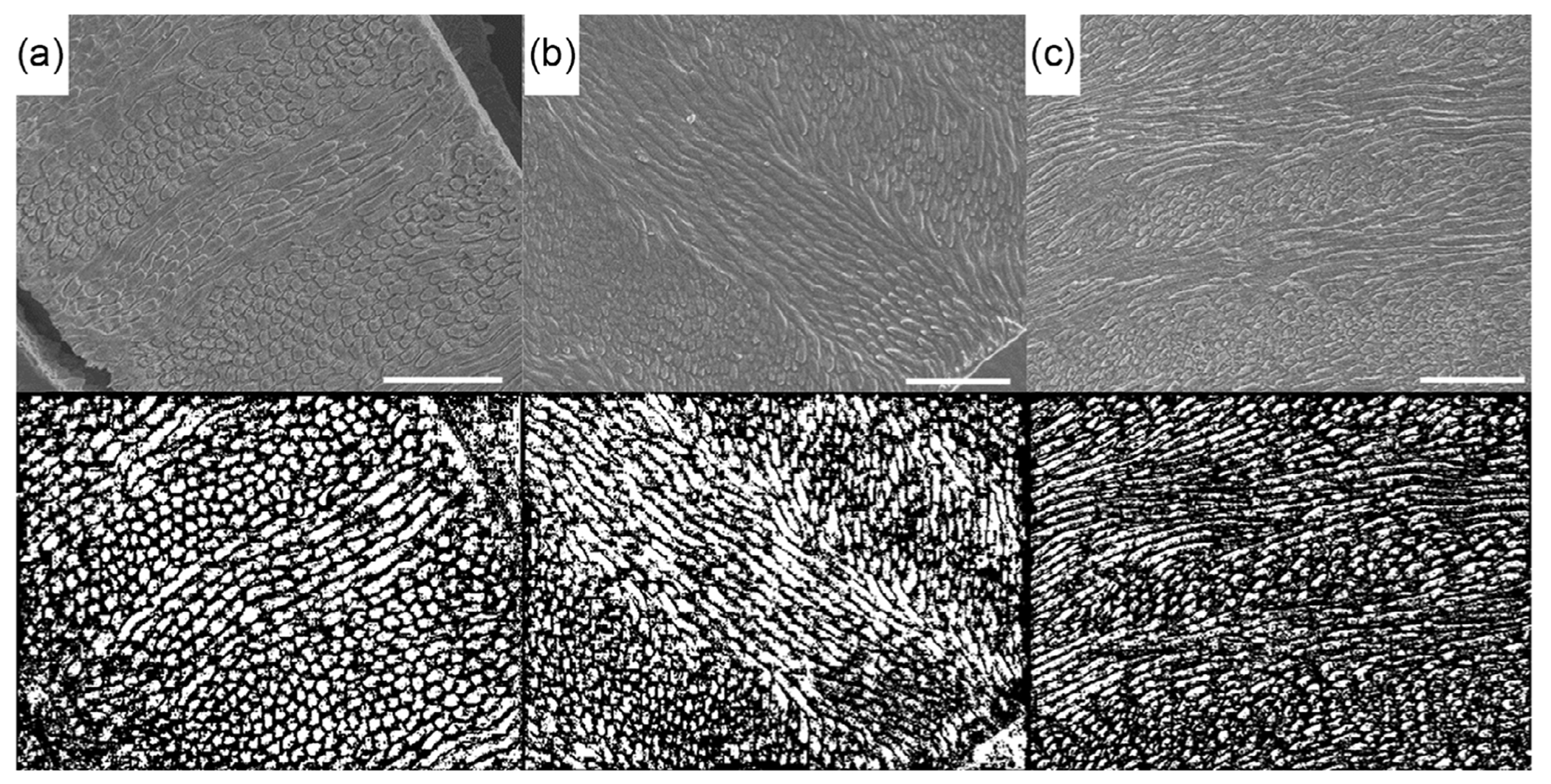
Comparison of the raw input image (top) and the segmentation product (bottom) for a) a 569x magnification image from a wild African dog tooth, b) a 500x magnification image from a snow leopard tooth, and c) a 500x magnification image from a raccoon tooth. Scale bar =50 μm. The comparisons involve a sample size of a single (*n* = 1) image for each species, which do not support probability value estimation or statistical test.

To allow the segmentation algorithm to accept images with a different magnification, the image was first cropped or padded with black (empty) pixels to make the image scale proportionate to that of a 500x image. The pixel density (resolution) was then corrected to that of a 500x image by resizing the resulting cropped or padded image to that of the 500x images. As a result, the rods were of the same pixel dimension as in the 500x image, but all images were 640 × 520 pixels. Figure 7 shows examples of segmentation maps produced from this testing. As shown, comparable segmentation could be achieved for these additional species, which were all of sufficient etch quality. Etching of the enamel preferentially removes material in regions of lower crystal packing density and helps accentuate the rods from the interrod regions. In general, the quality of etching across images and species can vary greatly, which has a substantial effect on the ability to distinguish rod features that are evident in SEM. This is primarily a function of the preparation technique, which is susceptible to slight changes in many processing variables.^[47]^ The enamel of some species is also more easily etched and imaged than others. Human enamel is comparatively difficult to achieve good etch and image quality with respect to that of other mammals. However, if an image of sufficient quality can be captured, this segmentation algorithm has been shown capable of segmenting the image invariant of the species and the minor variations in the enamel patterns present.

Results of the segmentation testing across image magnifications showed that the approach is robust provided the correct resizing has been applied. Images with magnifications ranging from 400x to 600x could be segmented without issues. It is possible that even higher-magnification images are acceptable if multiple images were stitched together to achieve an adequate field of the enamel structure. An image of 1000x magnification captures one-quarter of the area of an image with 500x magnification, which results in images that contain only 10–20 rods. Pilot experiments performed with that rod count showed that is too few for the segmentation algorithm to accurately determine the enamel pattern and segment rod objects. Preservation of the image scale is critical for this method since VGG16 and other pretrained CNNs generate size-dependent feature maps and the subsequent RF classifier is tuned to pick up those specific sizes and patterns.^[33–36,44]^ If the scale of the pattern is changed, the resulting dataset behaves uniquely from that involved in training. Images with a magnification of 500x were chosen for this effort because they contained several parazone and diazone bands in one image with an array of rods in each one with similar pitch angles. The microstructural parameter of primary interest in this investigation was rod pitch and hence the image sizes were selected accordingly. The image size could be adjusted as needed according to the feature of interest.

While this work focuses on the pitch angle of the enamel rods, the are other features that are of interest to the biomimetic community. For example, enamel rod diameter and decussation bandwidth are features that could be analyzed using a modified version of the proposed machine vision approach. However, to optimize the feature extraction and subsequent analysis, SEM imaging needs to be tailored to the feature of interest. To analyze enamel rod diameter via machine learning, higher-magnification images (mag >500x) such as that in Figure 1c should be used to train the CNN. Conversely, a lower magnification should be used to analyze bandwidth as it is a larger feature and requires a larger field of view. Nonetheless, this approach demonstrates a blueprint for machine learning-assisted feature extraction and analysis of enamel microstructure.

There are various limitations to the investigation that are important to highlight. Perhaps most important to note is the relatively low number of images that were used for training the algorithm. That was a deliberate choice due to the tedious nature of manually segmenting SEM images to establish ground truth data. Fortunately, results from the assessment of accuracy showed that the three training images were adequate to evaluate rod pitch angle across species. Another related limitation was the use of enamel from only two species, that is, bear and lion, for the ground truth. Although the rod diameters are approximately the same across the present species, anecdotal observations suggest that the rod shape and extent of interrod enamel are not the same. Those differences may result in the loss of some accuracy in quantifying the rods and their orientations. One concern of importance is that the evaluation of pitch from the rod shapes does not differentiate between negative and positive values. Additional work is underway to capture that information. Finally, the SEM images were obtained near the DEJ of cross sections of rods evident from the buccal-lingual section. Hence, the algorithm is not necessarily applicable for characterizing rod orientations on other section planes. Extending the algorithm to be capable of characterizing rods on other planes is possible and reserved for future work. Despite these limitations, the results of this investigation have shown the capabilities of a machine learning segmentation method for characterizing the enamel rod distributions from SEM images. The approach can support rapid quantitative description of the spatial distribution in enamel rods for detailed characterization of the microstructure, which is a critical step in the design of enamel-mimetic structures.

## 5. Conclusion

The application and validation of a CNN with RF classifier automatic segmentation algorithm to enamel SEM images was described. The approach was found capable of segmenting enamel images to extract quantitative information about the rod orientation in 3D. Specifically, the pitch angles of the enamel rods were quantified and compared to the values produced from a manually segmented ground truth image with accuracy approaching 80%. Furthermore, the developed model was found to be sufficiently generalized to be able to segment enamel images from species and magnifications that were not present in the original training dataset, provided appropriate image resizing is conducted. With systematic SEM imaging and recording of the specific coordinates of those images with respect to each other, the rod orientations as a function of distance through the enamel thickness and location within a tooth can be rapidly determined. While subject to some modest limitations, this approach will enable robust quantification of the enamel microstructure in the future, which was not possible previously.

## Supplementary Material

Supp Materials

Supporting Information

Supporting Information is available from the Wiley Online Library or from the author.

## Figures and Tables

**Table 1.** Comparison of the calculated pitch values for select rods in the algorithm segmented and ground truth images.

Rod Label	Pitch [°]	
	Algorithm Segmented	Ground Truth
Rod 1	25.0	39.6
Rod 2	40.6	39.1
Rod 3	12.9	11.9
Rod 4	18.6	23.4
Rod 5	13.6	12.9
Rod 6	49.0	40.5
Rod 7	52.9	59.0

## Data Availability

The data that support the findings of this study are available from the corresponding author upon reasonable request.
