# Supplementary material for: A Machine Learning Approach to Quantitative Analysis of Enamel Microstructure from Scanning Electron Microscopy Images
Source: Small Struct. Author manuscript; Available in PMC 2026 Apr 24. (PMC13105305; doi:10.1002/sstr.202400510)
Supplement: Supp Materials [file NIHMS2056992-supplement-Supp_Materials.pdf]

A Machine Learning Approach to Quantitative Analysis of Enamel Microstructure from SEM Images

Supplemental Information

The ‘optimum’ number of CNN layers for this study was identified to be five and those specific layers were identified to be (1) Block 1, Layer 1, (2) Block 1, Layer 2, (3) Block 2, Layer 1, (4) Block 2, Layer 2, and (5) Block 3, Layer 3. Furthermore, of the 10 best layer combinations (as distinguished by the accuracy), all layer combinations included some layers from Blocks 1, 2, and 3, with various additional layers from deeper blocks included intermittently. Hence, the most important features for the segmentation of complex microstructures are the shallower layers within the CNN. This is not particularly surprising since early CNN layers are associated with the capture of small-scale image textures, while deeper layers are associated with higher-order patterns within the image.[1,2]

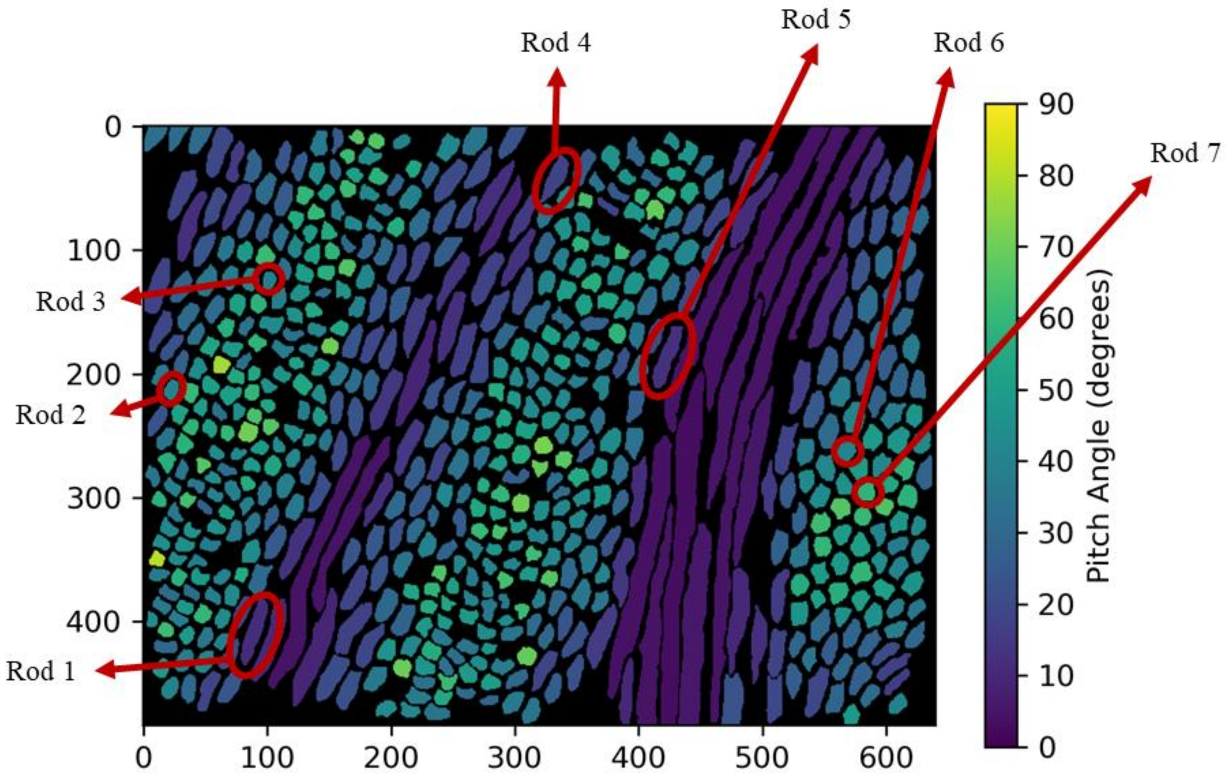

Supplementary Figure 1: Diagram showing the location of the rods compared in Table 1 as indicated in the manually segmented mask.

References

[1] E. Saraee, M. Jalal, M. Betke, *Comput. Vis. Image Underst.* **2020**, *195*, 102949.

[2] R. Bostanabad, *Comput.-Aided Des.* **2020**, *128*, 102906.
